# Role of CD47 in tumor immunity: a potential target for combination therapy

**DOI:** 10.1038/s41598-022-13764-3

**Published:** 2022-06-13

**Authors:** Jing Huang, Fangkun Liu, Chenglong Li, Xisong Liang, Chuntao Li, Yuanyuan Liu, Zhenjie Yi, Liyang Zhang, Siqi Fu, Yu Zeng

**Affiliations:** 1 Psychiatry Department and Mental Health Institute of the Second Xiangya Hospital, Central South University, Changsha, Hunan China; 2 National Clinical Research Center on Mental Disorders and National Technology Institute on Mental Disorders, Changsha, Hunan China; 3 Department of Neurosurgery, Xiangya Hospital, Central South University, 87 Xiangya Road, Changsha, 410008 Hunan China; 4 National Clinical Research Center for Geriatric Disorders, Xiangya Hospital, Central South University, 87 Xiangya Road, Changsha, 410008 Hunan China; 5 Department of Dermatology, The Second Xiangya Hospital, Central South University, 72 Renmin Road, Changsha, 410008 Hunan China

**Keywords:** Cancer, Computational biology and bioinformatics, Immunology, Biomarkers, Oncology

## Abstract

CD47 performs a vital function in cancer therapy by binding to different SIRPα, thrombospondin 1, and integrin. However, its role in tumor immunity and its correlation with prognosis among many cancer types remain unknown. The raw mRNA expression data of CD47 in cancer patients was downloaded from TCGA and GTEx datasets. The protein expression of CD47 was detected using a microarray. Kaplan Meier analysis and forest plot were performed to compare the effects of high and low expression of CD47 on overall survival in different cancers. In addition, the correlations between CD47 expression and immune cell infiltration, stromal components, immune checkpoint genes, tumor mutational burden (TMB), and microsatellite instability (MSI) were analyzed from the public database. The gene function was determined by Gene Set Enrichment Analysis (GSEA). The expressions of CD47 in CHOL, COAD, ESCA, HNSC, KIRC, STAD, and THCA were higher compared with normal tissues. Elevated expression of CD47 predicted poor prognosis in ACC, KICH, KIRP, LGG, PAAD and UCEC. CD47 expression was strongly associated with immune infiltrating cells among KICH, KIRP, LGG, and PAAD. In addition, significant positive correlations with most immune checkpoint genes including PDCD 1 (PD-1), CD274 (PD-L1), CTLA4 in BLCA, DLBC, KICH, KIRC, LUAD, LUSC, PAAD, PCPG, SKCM, STAD, UCEC, and UVM was noted for the expression of CD47. GSEA analysis demonstrated that CD47 was a key regulator in metabolism-related pathways. These findings provide novel evidence that CD47 could be utilized as a promising prognostic biomarker and combination treatment target in various cancers.

## Introduction

CD47 is a 47 kDa transmembrane protein, also known as integrin-related protein (IAP), composed of a short C-terminal intracellular variable spliced tail, five transmembrane helices, and an N-terminal extracellular IgV domain^1–3^. CD47 is a highly glycated protein distributed on the surface membrane of several cells, including red blood cells and non-hematopoietic cells, but its expression levels varied by cell types^2,4,5^. It is reported to mediate immune homeostasis and cell proliferation, migration, phagocytosis, and apoptosis. CD47 exerts these functions by binding to different ligands, including SIRPα, thrombospondin 1 (thrombospondin1, TSP-1), and integrin^6–8^. SIRPα, also known as protein tyrosine phosphatase substrate 1 (SHPS-1), is mainly expressed on myeloid cell membranes such as macrophages, myeloid dendritic cells, and monocytes; TSP-1 is a glycoprotein of extracellular matrix, composed of binding extracellular matrix components and cell surface receptors, secreted by macrophages, monocytes, platelets and a variety of non-hematopoietic cells^9,10^. Usually, CD47 appears as a "don't eat me" signal on healthy cells and could prevent phagocytosis by macrophages, whereas it is down-regulated on old and redundant cells which promotes the macrophages to remove them. CD47 on the cell surface is overexpressed in various cancer cells to help avoid the killing by innate immune cells.

In the 1980s, Knauf et al.^11^ first discovered the expression of CD47 on ovarian cancers, subsequently, a series of studies have confirmed that CD47 is overexpressed on tumor cells compared with healthy cells, including acute lymphoblastic leukemia cells^12^, lymphoma cells^7^, non-Hodgkin’s lymphoma (NHL) cells^13^, glioblastoma^14^, myeloma cells^15^, bladder tumor cells^16^, head and neck squamous cell carcinoma cells^17^, breast^18^, pancreas^19^, liver^20^, lung^21^, and prostate cancer cells^22^. Additionally, a few studies found that poor prognosis correlated with increased expression of CD47 in oral squamous cell carcinoma^23^, nasopharyngeal carcinoma^24^, triple-negative breast cancers^25^, ovarian carcinoma^26^, and non-small cell lung cancer^27^. These data demonstrated CD47 is of vital importance in cancer progression. Brightwell et al. found that epithelial ovarian cancer patients with low expression of CD47 had a better treatment response than standard therapy as well as extended OS^28^. Reseachers have also demonstrated that a higher CD47 expression was associated with poor prognosis in clear cell renal cell carcinoma^29^, non-small-cell lung cancer^27^, triple negative breast cancer^25^, oral squamous cell carcinoma^30^, and hepatocellular carcinoma^20^. Consequently, CD47 predicted poor prognosis in a couple of cancers, however, its prognosis value in many other cancer types need to be investigated.

Mounting evidence demonstrates that inhibition of the SIRPa–CD47 innate immune checkpoint is an effective and novel way for cancer therapy^9^. CD47 is a novel potent immunotherapy target in malignancies, targeting CD47 may be a novel strategy for cancer immunotherapy. A variety of studies have verified the synergistic effect of anti-CD47 combinated with target therapy^13,31^, chemotherapy^32^, and radiotherapy^33^. One of the most exciting and promising combination is targeting CD47 and other immune checkpoints. Liu et al.^34^ found that dual blockade of both CD47 and PD-L1 improved therapeutic efficacy versus monotherapy by increasing DNA sensing, DC cross-presentation, and anti-tumor T cell response. Bispecific antibody targeting CD47 and PD-L1 could maximize antitumor immunity by activating type I interferon pathways, increasing antigen presentation in dendritic cells, macrophage, and Tcf7+ stem-like progenitor CD8 T cell populations^35^. Targeting CD47 in combination with PD-1 may potentially improve the outcomes of patients with anaplastic thyroid carcinoma^36^. Similarly, Dual targeting CD47 and CTLA-4 promotes immunity against colon cancers^37^. Anti-CD47 targeting in pancreatic cancer could remodel tumor- infiltrating immune cells^38^ and enhance CD19/CD3-bispecific T cell engager antibody-mediated lysis of B cell malignancies^39^. However, limited is known about the combination treatments of anti-CD47 and other immune checkpoint blockers except for PD1/PDL1/CTLA4.

In this study, we performed a pan-cancer analysis between CD47 expression and patients’ prognosis. We further investigated the correlation of CD47 expression with the level of immune infiltration across a large variety of cancers, and explored whether immune checkpoint genes correlated with CD47 expression in 33 cancer types. Lastly, we enriched the functional pathways CD47 which may be involved in cancers. Our findings provide new insights linking CD47 with numerous tumor prognoses, effects and mechanisms of targeting CD47 in anti-tumor immunity. Of note, combination treatment of anti-CD47 and immune checkpoint inhibitors may provide new approaches and improvements for anti-cancer strategies.

## Materials and methods

### Raw data acquisition

We downloaded transcriptome RNA-seq data of 33 cancers over 10,000 patients from TCGA (The Cancer Genome Atlas) database. The cancer types were listed in Supplement Table 1.

### Analysis of CD47 expression and survival analysis in cancers

The data of CD47 expressions in both tumor and matched normal tissue were extracted from Genotype Tissue Expression (GTEx) and TCGA datasets. We used log2 (TPM+1) transformed expression data for plotting. Kaplan Meier analysis by log-rank test was applied to compare the overall survival (OS) for patients stratified based on the median gene expression level of CD47. Univariate Cox model was used to investigate the relationship between gene expression levels and patient survival in various cancers. *P* < 0.05 was considered as a statistical difference.

### Relationship between the expression of CD47 and immunity

To better understand the relation between CD47 and immunity, we analyzed the correlation between the proportion of the tumor infiltration cells and the expression of CD47 by TCGA datasets, such as B cells, CD8+ T cells, CD4+ T cells, dendritic cells (DCs), macrophages, and neutrophils. Additionally, Stroma Score and ESTIMATE Score were utilized to assess tumor purity and stroma cell proportion^40^. Furthermore, we explored the relationship between CD47 expression and immune checkpoint genes, Tumor mutation burden (TMB) as well as Microsatellite instability (MSI) analysis regarding the relationships were investigated by Sangerbox.

### Biofunctional analysis

The data was divided into two groups according to the level of CD47 expression, then enrichment analysis was performed by KEGG and HALLMARK depending on expression levels of CD47. Gene sets with |NES| > 1, NOM p-val < 0.05, and FDR q-val < 0.25 were determined as significantly enriched.

### Statistical analysis

The Student’s t-test was utilized to investigate the gene expression. Spearman’s correlation analysis was used to evaluate the CD47 expression with stroma cell proportion score or immune cell infiltration score or immune checkpoint genes score. All analyses were performed with the R software (version 3.6.1). *P* < 0.05 was considered as a statistical difference.

### Ethical approval and ethical standards

The study was approved by the ethics committee of the Xiangya Hosptial, Central South University. All the methods and protocol with human data were performed in accordance with ethical standards of the ethics committee and the Helsinki Declaration of 1975 and its later amendments.

### Informed consent

Informed consent was obtained in all cases for the tissue microarray.

## Results

### The different mRNA expression of CD47 across cancers

**Figure 1:**
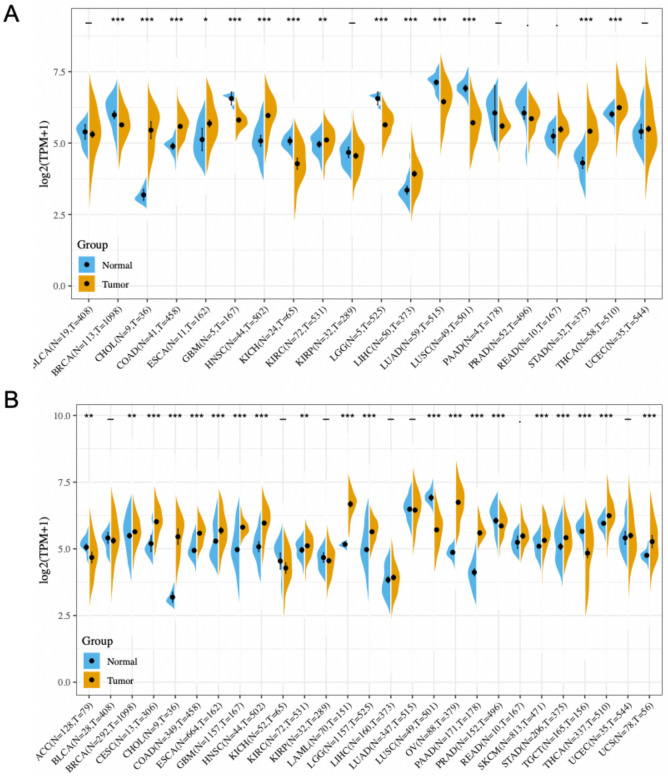
The expression of CD47 across a large variety of cancers. (**A**) The differential expression of CD47 between twenty types of cancer tissues and their counterparts were analyzed by the TCGA database and (**B**) integrated analysis of twenty-seven cancer types by TCGA and GTEx datasets. CD47 was high expressed in tumor samples of CHOL, COAD, ESCA, HNSC, KIRC, STAD, and THCA, and low expressed in tumor samples of LUSC than normal tissues in both comparisions. *Indicates *P* value < 0.05; ** indicates *P* value < 0.01; *** indicates *P* value < 0.001.

To understand the relationship between CD47 and various cancers, we explored the RNA expression level of CD47 in different cancers and matched normal tissues. Initially, we evaluated CD47 expression level between the tumor and matched normal tissues across twenty cancer types via the TCGA database (Fig. 1A), and then verified by twenty-seven cancer types using the integrated database from GTEx and TCGA datasets (Fig. 1B). CD47 was consistently highly expressed in tumor samples of CHOL, COAD, ESCA, HNSC, KIRC, STAD, THCA, and consistently low expressed in tumor samples of LUSC than normal tissues in both comparisons.

### High expression of CD47 predicts worse prognosis in a couple of cancers

**Figure 2:**
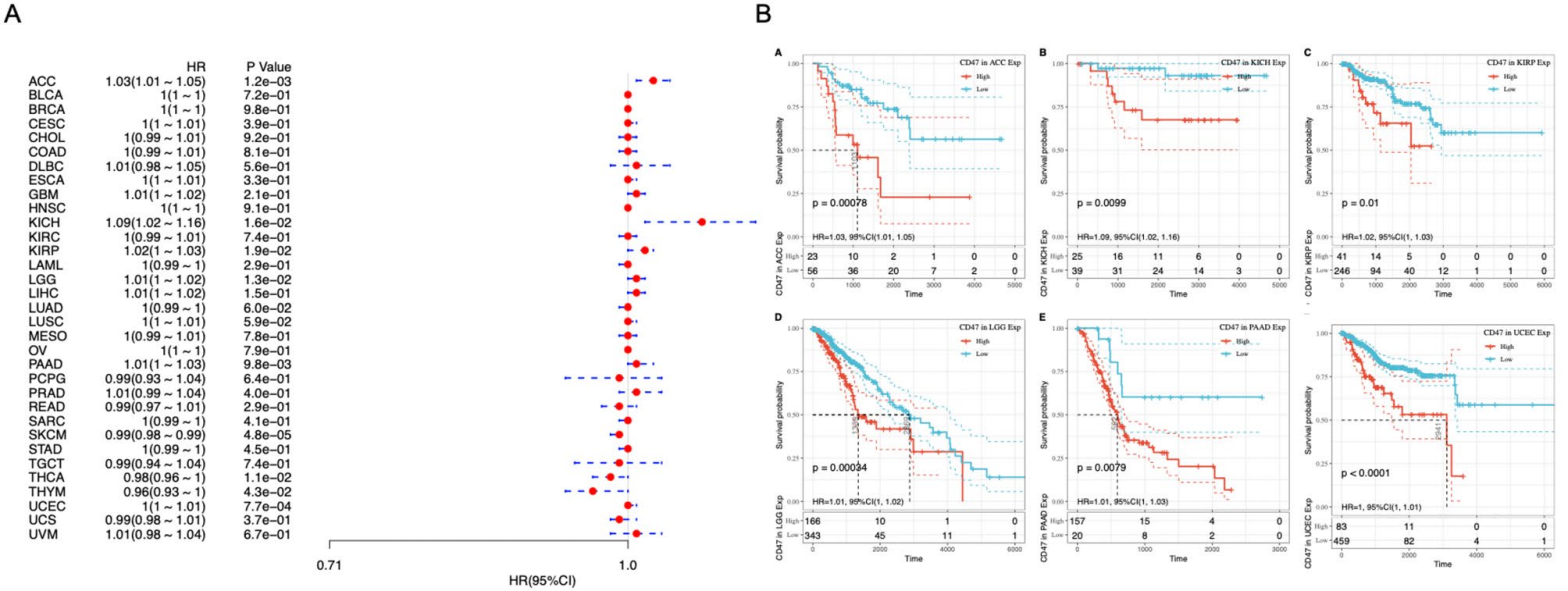
High expression of CD47 was associated with a worse prognosis in cancers. (**A**) Forest plot was performed to analyze CD47 expression on OS in 33 cancer types by Cox regression model. (**B**) Kaplan–Meier analysis demonstrated high expression of CD47 predicted poor prognosis in ACC, KICH, KIRP, LGG, PAAD, UCEC.

To further evaluate the prognostic value of CD47 among different cancer types, Forest plot was performed to analyze the expression of CD47 on OS across 33 cancer types by Cox regression model. It was indicated that higher expression of CD47 predicted poor survival in ACC, KICH, KIRP, LGG, PAAD, and UCEC, while associated with favorable prognosis in SKCM, THCA, and THYM (Fig. 2A). Six of the cancer types show a significantly increased hazard ratio, whereas three of them significantly decreased hazard ratios with higher CD47, and others show no significance. In addition, Kaplan–Meier survival analysis demonstrated that high expression of CD47 correlated with unfavorable prognosis in ACC (HR = 1.03, *P* < 0.001), KICH (HR = 1.09, *P* = 0.010), KIRP (HR = 1.02, *P* = 0.01), LGG (HR = 1.01, *P* < 0.001), PAAD (HR = 1.01, *P* = 0.008), UCEC (HR = 1.01, *P* < 0.001) (Fig. 2B). High expression of CD47 predicted poor prognosis in ACC, KICH, KIRP, LGG, PAAD and UCEC based on both analyses.

### The relationship between CD47 expression and immune infiltrating level across various cancers

**Figure 3:**
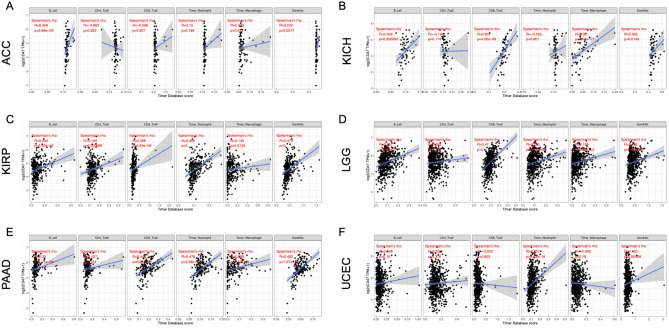
The relationship between CD47 expression and immune infiltrating level in ACC, KICH, KIRP, LGG, PAAD, UCEC (**A**–**F**). The expression of CD47 is positively related to the tumor infiltrating cells such as CD4+ T cells, CD8+ T cells, B cell, neutrophil, dendritic cells and macrophages in various types of cancer.

The immune cells are of vital importance to patients with diverse cancers to modulate tumor invasion and aggression. Increased tumor infiltrating lymphocytes are valuable prognostic and predictive index for response of immunotherapy and prognosis of cancer patients. Consequently, we investigated the correlation between CD47 expression and immune infiltration in a variety of cancers by TIMER database^41^. The results demonstrated that CD47 levels significantly correlated with the infiltration levels of B cells in six cancer types including ACC (r = 0.458, *P* < 0.001), KICH (r = 0.402, *P* < 0.001), KIRP (r = 0.246, *P* < 0.001), LGG (r = 0.287, *P* < 0.001), PAAD (r = 0.33, *P* < 0.001), UCEC (r = 0.056, *P* < 0.001); CD4+ cells in one cancer type that is KIRP (r = 0.194, *P* < 0.001); CD8+ T cells in four cancer types including KICH (r = 0.631, *P* < 0.001), KIRP (r = 0.266, *P* < 0.001), LGG (r = 0.47, *P* < 0.001), PAAD (r = 0.445, *P* < 0.001); neutrophils in four cancer types types including KIRP (r = 0.465, *P* < 0.001), LGG (r = 0.235, *P* < 0.001), PAAD (r = 0.476, *P* < 0.001), UCEC (r = 0.337, *P* < 0.001); macrophages in four cancer types including KICH (r = 0.58, *P* < 0.001), KIRP (r = 0.145, *P* < 0.001), LGG (r = 0.168, *P* < 0.001), PAAD (r = 0.299, *P* < 0.001); and dendritic cells in six cancer types including ACC (r = 0.242, *P* = 0.032), KICH (r = 0.302, *P* = 0.015), KIRP (r = 0.473, *P* < 0.001), LGG (r = 0.162, *P* < 0.001), PAAD (r = 0.482, *P* < 0.001), UCEC (r = 0.123, *P* = 0.004) (Fig. 3). The above data also revealed that the expression levels of CD47 was strongly associated with immune infiltrating cells in KICH, KIRP, LGG, and PAAD cancers.

### Correlation between CD47 expression and tumor purity in cancers

**Figure 4:**
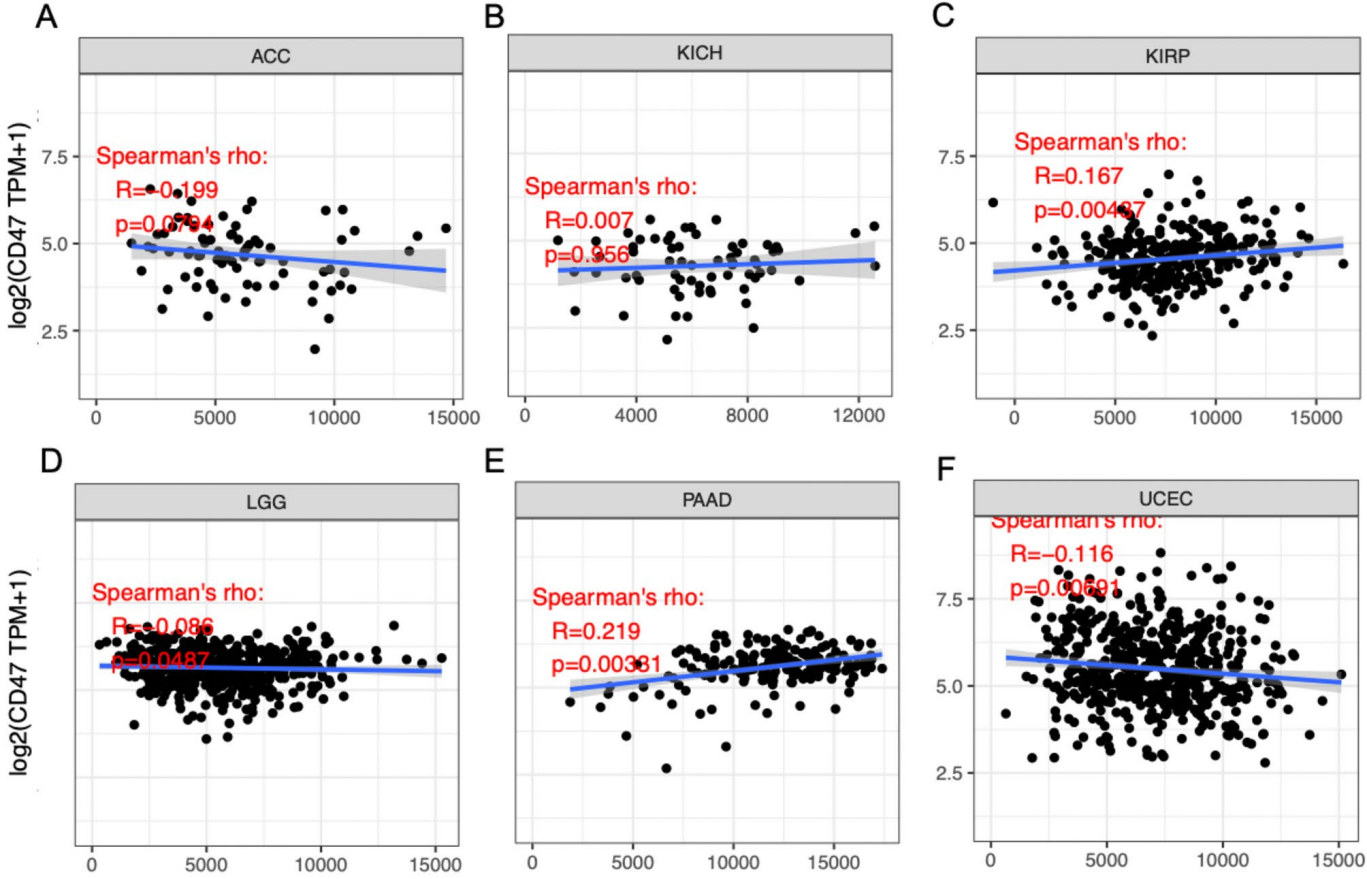
Correlation of CD47 expression with Estimate Score in ACC, KICH, KIRP, LGG, PAAD, UCEC (**A**–**F**). The level of CD47 expression was inversely related to tumor purity in LGG, and positively correlated with tumor purity in KIRP, PAAD.

To explore the relationship between CD47 and tumor purity of cancers, we analyzed the ESTIMATE Score. In a couple of cancer types, the level of CD47 expression was inversely related to tumor purity in LGG (r =  − 0.086, *P* = 0.049) and UCEC (r =  − 0.116, *P* = 0.007). However, it was positively correlated with tumor purity in KIRP (r = 0.167, *P* = 0.004) and PAAD (r = 0.219, *P* = 0.004) (Fig. 4).

### Correlation between CD47 expression and stroma score in cancers

**Figure 5:**
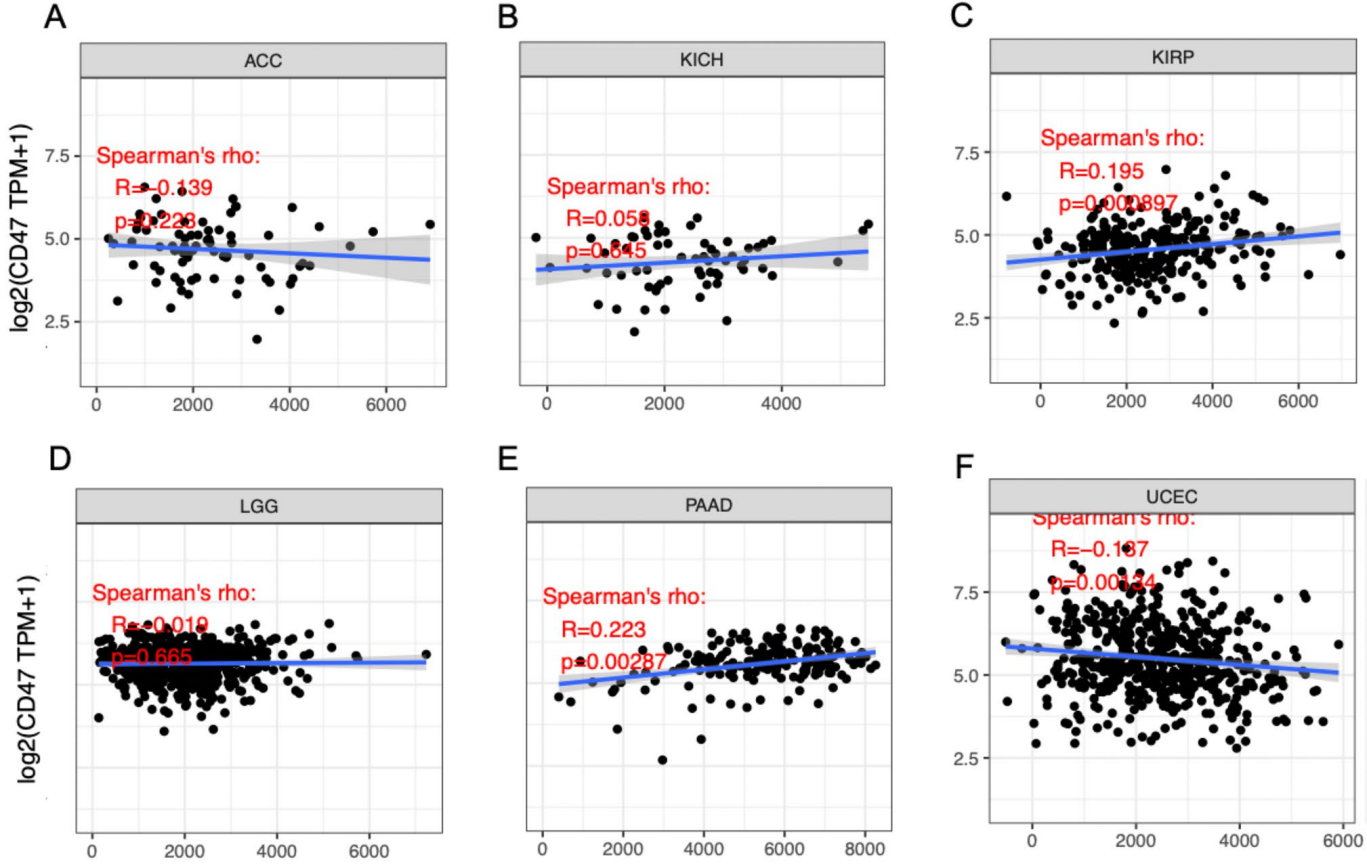
Correlation of CD47 expression with Stromal Score in ACC, KICH, KIRP, LGG, PAAD, UCEC (**A**–**F**). The level of CD47 expression was negatively associated with tumor purity in UCEC, and positively related to tumor purity in KIRP, PAAD and no significance in ACC, LGG, and KICH.

To figure out the relation between CD47 and stroma cells of cancers, we analyzed the STROMA Score. CD47 expression was negatively associated with tumor purity in UCEC (r =  − 0.187, *P* = 0.001). However, it was positively related to tumor purity in KIRP (r = 0.195, *P* < 0.001), PAAD (r = 0.223, *P* = 0.003) (Fig. 5). Taken together, KIRP and PAAD were in positive correlation with StromalScore, and ESTIMATEScore. Conversely, UCEC was negatively correlated with these two scores. Consequently, Those data indicated that the expression level of CD47 was closely linked to the extent of tumor immune infiltration among cancers in both favorable and unfavorable cancers.

### Correlations between CD47 expression and immune checkpoint markers in cancers

**Figure 6:**
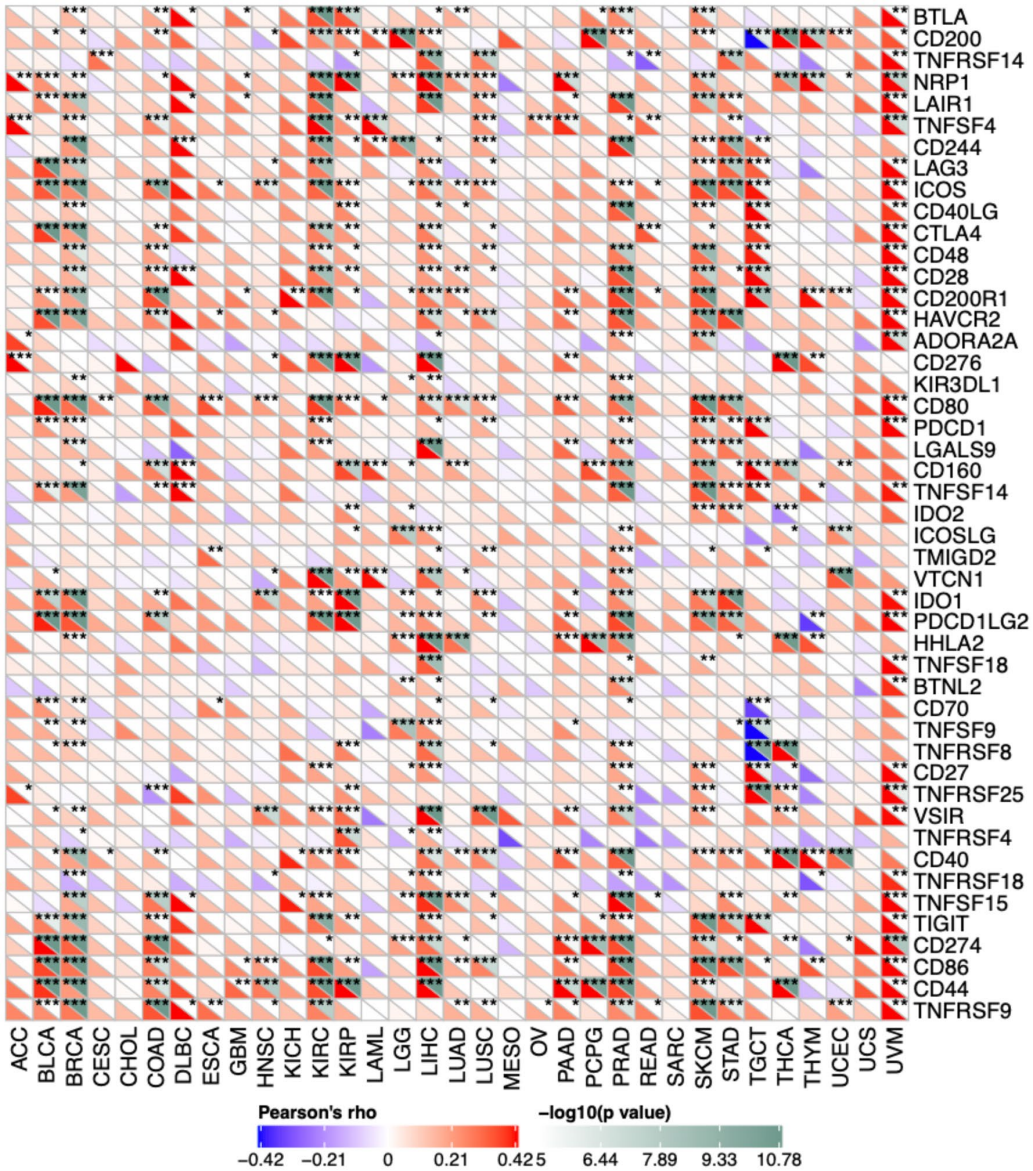
The pearson analysis between CD47 expression and immune checkpoint genes in 33 cancer types. The expression of CD47 was positively correlated with immune checkpoint markers in BLCA, DLBC, KICH, KIRC, LUAD, LUSC, PAAD, PCPG, SKCM, STAD, UCEC and UVM. and Increased CD47 was positively correlated with BTLA, ICOS, CD40LG, CTLA4, CD48, CD28, CD200R1, HAVCR2, CD80, PDCD1(PD-1), CD160, IDO1, HHLA2, CD40, TNFSF15, TIGIT, CD274, CD86, CD44, TNFRSF9. *Indicates *P* value < 0.05; ** indicates *P* value < 0.01; *** indicates *P* value < 0.001.

Increasing evidence has demonstrated that immune checkpoint genes, including PD-1, PDL1, and CTLA-4, have played important roles in immune evasion. To further explore the correlation between CD47 expression and the extent of immune infiltration, we investigated the association between CD47 and the expression of 47 immune checkpoint genes among 33 cancer types. As indicated in Fig. 6, the expression of CD47 was positively correlated with immune checkpoint markers in the majority of cancer types including BLCA, DLBC, KICH, KIRC, LUAD, LUSC, PAAD, PCPG, SKCM, STAD, UCEC, and UVM. In addition, increased CD47 was positively correlated with BTLA, ICOS, CD40LG, CTLA4, CD48, CD28, CD200R1, HAVCR2, CD80, PDCD1(PD-1), CD160, IDO1, HHLA2, CD40, TNFSF15, TIGIT, CD274(PD-L1), CD86, CD44, and TNFRSF9 across 33 cancers (Fig. 6).

### Correlations between CD47 expression and TMB as well as MSI in cancers

**Figure 7:**
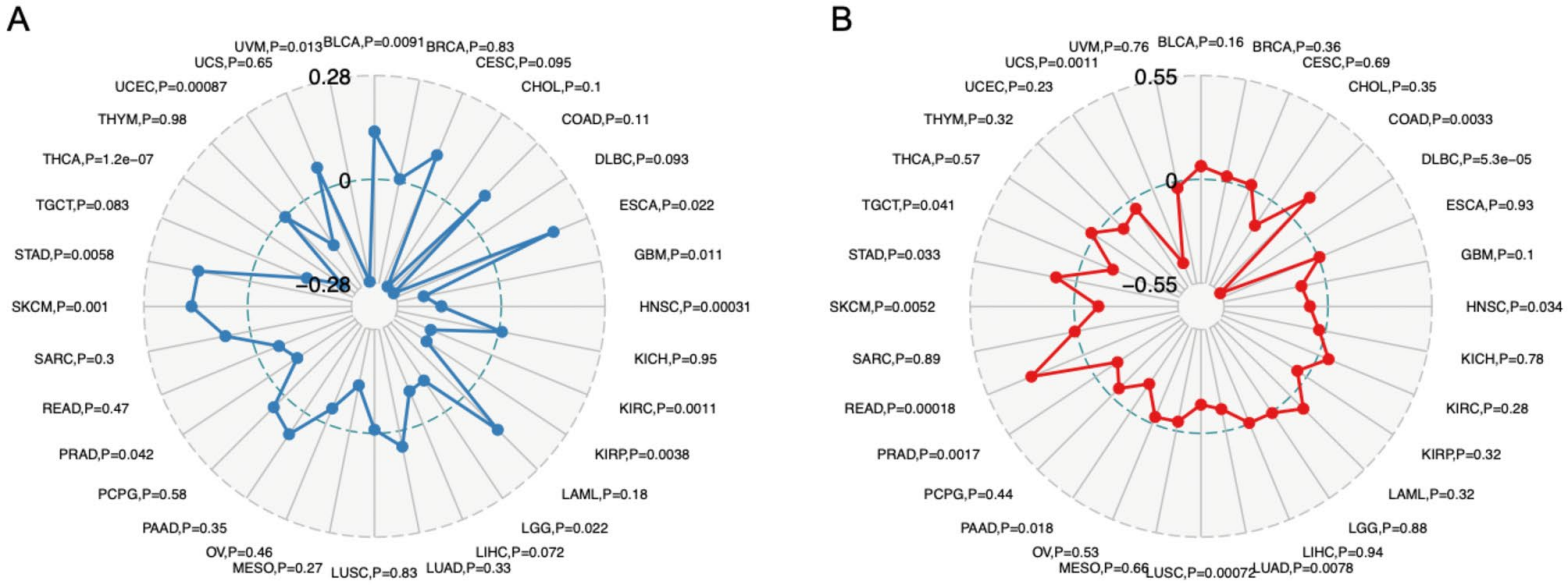
Radar map plotting showed the relationship between CD47 expression and TMB (**A**), CD47 expression and MSI (**B**) in 33 cancer types. (**A**) Increased expression of CD47 was positively associated with TMB in BLCA, ESCA, SKCM, STAD, while negatively correlated with GBM, HNSC, KIRC, KIRP, LGG, PRAD, THCA, UCEC and UVM. (**B**) Increased expression of CD47 was positively related to COAD, READ, STAD,and negatively associated with DLBC, HNSC, LUAD, LUSC, PAAD, PARD, SKCM, TGCT, and UCS.

We further explored the correlations between CD 47 expression and TMB and MSI, which were valuable markers for tumor immunotherapy and prognosis. The expressions of CD47 and TMB were significantly different among various cancer types. Increased expression of CD47 was positively associated with TMB in BLCA, ESCA, SKCM, STAD, while negatively correlated with GBM, HNSC, KIRC, KIRP, LGG, PRAD, THCA, UCEC and UVM (Fig. 7A). As far as MSI was conferred, elevated expression of CD47 was positively related to COAD, READ, STAD, while negatively associated with DLBC, HNSC, LUAD, LUSC, PAAD, PARD, SKCM, TGCT, and UCS (Fig. 7B).

### Functional enrichment analysis of CD47 in cancers

**Figure 8:**
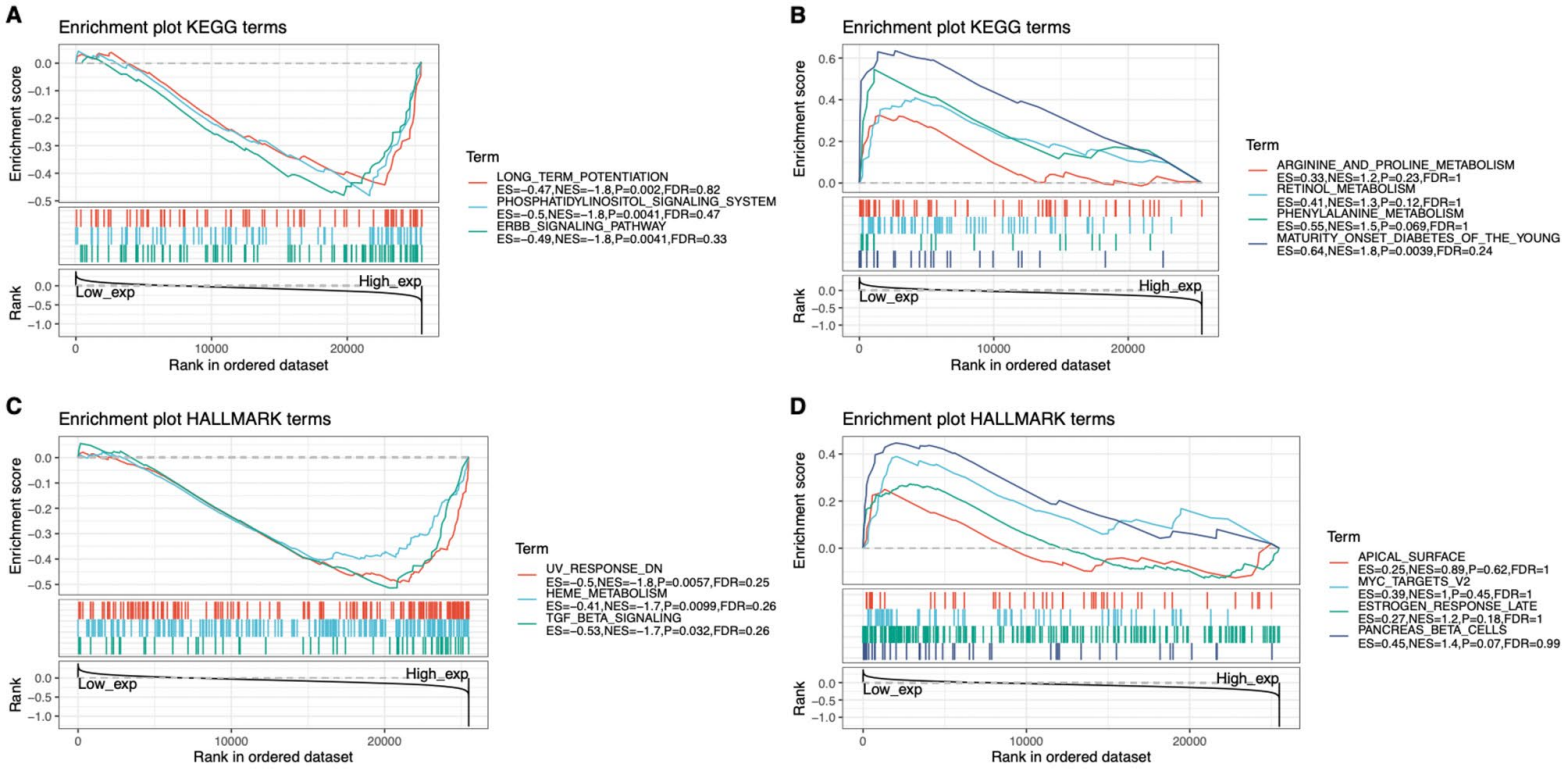
Biofunctional analysis of CD47 across cancers. KEEG terms showed top three negative enrichment gene sets (**A**) and top three positive ones (**B**). HALLMARK terms demonstrated top three negative enrichment gene sets (**C**) and positive ones (**D**) respectively. (**A**,**B**) High expression of CD47 was negatively associated with long-term potentiation, phosphatidylinositol signaling system and ERBB signaling pathway, and positively associated with arginine/proline/metabolism, retinol/metabolism, phenylalanine/metabolism. (**B**,**C**) Increased expression of CD47 was negatively linked to UV response, heme metabolism, TGF-beta pathway, and positively associated with the apical surface, MYC and estrogen response late.

To better understand the potential mechanisms of CD47 in cancers, functional enrichment analysis was explored based on the high and low expression of CD47 (Fig. 8). High expression of CD47 was negatively associated with long-term potentiation, phosphatidylinositol signaling system, and ERBB signaling pathway by KEEG analysis. On the other side, expression of CD47 was positively associated with arginine/proline/metabolism, retinol/metabolism, phenylalanine/metabolism. Furthermore, HALLMARK enrichment term suggested that increased expression of CD47 was negatively linked to UV response, heme metabolism, and TGF-beta pathway. Additionally, CD47 was positively associated with the apical surface, MYC and estrogen response late. All the above data demonstrated that high CD47 expression was positively involved in metabolism-related pathways.

## Discussion

CD47 played a vital role in cancer cell proliferation, invasion, and metastasis^9^. Notably, it was found to be a key regulator in immunosurveillance and immune evasion in a large variety of cancers^10^. Recent studies verified that using anti-CD47 antibodies or tumor cell/tumor antigen vaccines lacking CD47 could help mice to eliminate cancer cells in both solid and hematopoietic tumors^9^. CD47 has been identified to be an adverse prognostic factor among various cancers including gastric cancer^42^, oral squamous cell carcinoma^23^, nasopharyngeal carcinoma^24^, triple-negative breast cancers^25^, ovarian carcinoma^26^, and non-small cell lung cancer^27^, while high expression of CD47 was a favorable prognostic factor in cutaneous melanoma^43^. Similarly, the current study found that elevated expression of CD47 predicted unfavorable prognosis in ACC, KICH, KIRP, UCEC and favorable prognosis in PAAD, SKCM, THCA, and THYM. Those studies together with our pan-cancer analysis suggested CD47 was an important biomarker in various cancer types.

The tumor immune microenvironment is of great importance for cancer cell proliferation, metastasis, survival, and treatment resistance^44,45^. Immunotherapy has achieved prominent effects among the treatment of different cancers, such as monoclonal antibodies, chimeric antigen receptor (CAR) T cells, immune checkpoint inhibitors, cancer vaccines, therapeutic antibodies, and cell therapy^46,47^. As a novel immunotherapy target, to date, there are four major strategies of anti-CD47 treatment: (1) CD47 antibody can enhance cancer cell phagocytosis of macrophages by blocking the binding of CD47-SIRPα; (2) CD47 antibody or SIRPα-Fc fusion protein can also exert cytotoxic effects through the Fc segment; (3) CD47 antibody could directly induce tumor cells apoptosis via a caspase-independent mechanism; (4) CD47 antibody could work with prophagocytic molecules to make DC phagocytose tumor cells, and then present the antigen to T cells, thereby activating the adaptive immune response. Previous studies have also revealed that CD47 inhibition could promote CD8+ T cells for tumor cell killing and loss of CD47 in the tumor stroma increases the sensitivity of some tumors to standard therapy^48^. Anti-CD47 increased the pro-inflammatory macrophages and reduced the anti-inflammatory macrophages^38^. In the present study, we found CD47 was closely related to immune cells in various cancers. CD47 expression was positively associated with CD4+ T cell, CD8+ T cell, B cell, neutrophil, dendritic cell, and macrophage infiltration of numerous cancers. However, CD47 could play a different role among different cancers. Although loss or inhibitory of CD47 usually enhanced eliminating cancer cells, CD47 could positively and negatively regulate NK cell function in human melanomas, which may explain CD47 was associated with improved survival in melanomas. In addition, increased expression of CD47 was positively associated with TMB in BLCA, ESCA, SKCM, STAD, while negatively correlated with GBM, HNSC, KIRC, KIRP, LGG, PRAD, THCA, UCEC, and UVM. As far as MSI was conferred, elevated expression of CD47 was positively related to COAD, READ, STAD, while negatively associated with DLBC, HNSC, LUAD, LUSC, PAAD, PARD, SKCM, TGCT, and UCS. TMB and MSI analysis also supported its different roles in different cancers. It seems high expression of CD47 was negative with TMB/MSI in several poor prognosis cancers, while positively with TMB/MSI in a few favorable cancers. Taken together, CD47 expression was extensively related to cancer immunity.

Furthermore, the level of CD47 was positively correlated with most immune checkpoint genes while also negatively with a few genes in pan-cancer analysis. Of note, previous studies have identified combination inhibition of CD47 and PD-L1 could lead to a synergistic inhibition effect on tumor growth in some pancreatic cancer cell lines^38^. Combination blockade of CD47 and PD1, CTLA4 also achieved better anti-tumor effect^34,37^. Furthermore, a couple of ongoing clinical trials are targeting dual immune checkpoints such as CD47/PD-1(HX009), CD47/CD137 (DSP107), CD47/PD-L1 (IBI322, PF-07257876), CD47/CD20 (IMM0306), CD47/CD19 (TG-1801)^49^. The present analysis found elevated expression of CD47 not only positively correlated with PD1, PD-L1, CTLA4, but also with multiple other immune checkpoints such as IDO1, CD40, CD160, CD86, CD44 across various cancer types. Consequently, the novel combination of CD47 and other immune molecules bcould provide new strategies for cancer treatment.

Additionally, biofunctional analysis by KEGG and HALLMARK analysis on CD47 through GSEA in the current study suggested high CD47 expression was mainly associated with metabolism-related pathways. Similary, Previous studies have found that activation of CD47 from both neutrophils and epithelial cells caused a shift of specific signaling elements from a tyrosine-phosphorylated to a tyrosine-dephosphorylated status by phosphatidylinositol 3-kinase and tyrosine kinases, which facilitated neutrophil migration across endothelial and epithelial monolayers^50^, which supported the effect of CD47 in phosphorylation. Other researchers found that CD47 deficiency could increas TGF-β1 expression by phosphorylation of the TGF-β1 receptor-regulated transcription factors SMAD-2 and SMAD-3^51^, which supported the effect of CD47 in TGF-β pathway. Additionally, researchers also demonsrated that CD47 blockade or deficiency broadly prevents CD47 regulating metabolic pathways required to control glucose metabolism, oxidative stress, DNA repair, and energetics after exposure to ionizing radiation^52^ and combination blockade of CD47 and HER2 achieved an antitumor immune response in radioresistant breast cancer^18^, which provide evidence for the combination of CD47 and ERBB signaling. Those studies together with our analysis demonstrated CD47 closely correlated with metabolism pathways like TGF-β, phosphorylation and so on. Therefore, the combination of anti-CD47 with other immune checkpoint blockades or a combination of anti-CD47 with metabolism regulators could shed light on novel cancer treatment.

Our study has some limitations. Firstly, the mRNA level of CDC47 is assessed in our study, its correlation with immune checkpoint inhibitors or metabolism regulators needed to be validate in future experiments. Secondly, we analyzed the possible pathways associated with CD47, the results of bioinformatic analysis needed to be verified in biological experiments for further validation. Further mechanistic studies will be beneficial to undermine the value of CD47 as a potential target of immunotherapy at the molecular and cellular levels.

In sum, all the data in the current study suggested the CD47 as an effective prognostic biomarker in various types of human cancers. Anti-CD47 treatment could be utiliezd as a promising strategy in a variety of cancers. Besides, our results shed light on the significant role of CD47 in tumor immunity and metabolic activity. However, more data are needed to clarify the mechanisms of CD47 in those activities. Future prospective studies will be focusing on the combination of anti-CD47 and immune checkpoint inhibitors or metabolism regulators that could provide an immuno-based strategy for cancer therapy.

## Supplementary Information

Supplementary Information.

## Data Availability

All datasets are available on reasonable request.
